# Molecular characterization of NDM and OXA-48-like-producing *Klebsiella pneumoniae* ST16 and hypervirulent ST337 clone among two patients; a case report

**DOI:** 10.1186/s12879-024-09762-7

**Published:** 2024-08-22

**Authors:** Sarvenaz Sokhanvari, Atiyeh Bagheri, Farzad Badmasti, Hamid Solgi

**Affiliations:** 1 Isfahan Endocrine and Metabolism Research Center, Isfahan University of Medical Sciences, Isfahan, Iran; 2 Department of Bacteriology, Pasteur Institute of Iran, Tehran, Iran; 3 Amin Hospital, Isfahan University of Medical Sciences, Isfahan, Iran

**Keywords:** Hypervirulent *Klebsiella pneumonia*, NDM and OXA-48-like-producing *Klebsiella pneumonia*, Multilocus sequence typing, VAP, UTI

## Abstract

Carbapenem-resistant *Klebsiella pneumoniae* (CRKP) infections are a major public health problem, requiring the use of last-resort antibiotics such as colistin. However, there is concern regarding the emergence of isolates resistant to this agent. The report describes two patients with urinary tract infection (UTI) and ventilator-associated pneumonia (VAP) infection caused by CRKP strains. The first case was a 23-year-old male with UTI caused by a strain of ST16 co-harboring *bla*_CTX-M_, *bla*_TEM_, *bla*_SHV_, *bla*_NDM_, *bla*_OXA-48-like_ genes. The second case was a 39-year-old woman with VAP due to hypervirulent ST337-K2 co-harboring *bla*_SHV_, *bla*_NDM_, *bla*_OXA-48-like,_ *iucA*, *rmpA2* and* rmpA*_._ The patients’ general condition improved after combination therapy with colistin** (**plus meropenem and rifampin, respectively) and both of them recovered and were discharged from the hospital. This study highlights the necessary prevention and control steps to prevent the further spread of CRKP strains should be a priority in our hospital.

## Introduction

Carbapenem-Resistance *Klebsiella pneumoniae* (CRKP) is a major global public health threat that can cause invasive hospital-acquired infections among patients and is therefore associated with high morbidity and mortality [1]. The rise of this pathogen has led to the resurgence of colistin as a last-resort therapy. Classic *K. pneumoniae* (cKPs) and hypervirulent *K. pneumoniae* (hvKPs) are two different variants of *K. pneumoniae*. hvKp is an evolving pathotype that is considered to be more virulent than classical cKp in both basic research and clinical research [2]. In recent years several studies have reported the existence and increasing prevalence of multidrug-resistant hvKP strains, especially extended-spectrum β-lactamase (ESBL)-producing hvKP and carbapenem-resistant hvKP [3, 4]. In Iran, due to the increase in the prevalence of carbapenemase-producing *K. pneumoniae* isolates, colistin consumption has increased significantly, which has already caused the emergence of colistin-resistant isolates [4, 5]. Here, we present the clinical and microbiological findings of two patients who developed infection caused by ST337-K2 carbapenemase-producing-hvKp and ST16 carbapenemase-producing infection. Both patients were treated successfully with colistin combination therapy and discharged from the hospital.

## Case presentation

### Case 1

On October 6, 2020, a previously healthy 23-year-old male patient was admitted to the emergency department following a fall from a height, resulting in multiple traumas. His initial vital signs were as follows: blood pressure of 131/82 mmHg, pulse of 90 bpm, respiratory rate 20 of breaths/min, and body temperature of 37 ℃. The patient, with no prior medical history, underwent intubation and was subsequently admitted to the intensive care unit (ICU) with a diagnosis of subdural hematoma and pleural effusion. After being admitted, he was empirically treated with intravenous ceftazidime (1 g IV every 8 h, 11 days) and clindamycin (600 mg IV every 8 h, 11 days). On the 5th day of admission, vancomycin was experimentally added to the antibiotic treatment by the infectious specialist with the suspicion of nosocomial infection. On the 12th day of his ICU stay, the patient was successfully extubated. He developed fever (39.5°C) and leukocytosis (18,200 per µL) on the 23rd day, and blood and urine were obtained for microbiological culture.

On day 26 of his ICU stay, the urine culture showed heavy growth of CRKP isolate (minimum inhibitory concentrations [MICs] 16 and 2 μg/ml for meropenem and colistin, respectively) that was also resistant to piperacillin/tazobactam, ceftazidime, cefepime, amikacin, gentamicin, ciprofloxacin, and levofloxacin. After acquiring susceptibility results, the combination antibiotic therapy was adjusted to colistin (9 million IU) divided into two daily doses and meropenem (1 g IV every 8 h). The patient was continued on meropenem and colistin for seven days. The patient was discharged from the hospital on day 33.

### Case 2

On May 16, 2021, a 39-year-old woman with a history of multiple sclerosis (MS) and psychiatric illness presented to the emergency department with complaints of progressive decrease in consciousness, weakness, arthralgia, abdominal distension, and tenderness in the right lower quadrant and left lower quadrant.

The patient underwent surgery for necrosis and gangrene of the small bowel, specifically a resection and ileostomy on May 16th. Due to positive findings on High-Resolution Computed Tomography, there was suspicion of COVID-19 pneumonia (Covid PCR negative). Pathological findings were also observed on brain CT, and the patient received treatment with remdesivir and methylprednisolone.

During the hospitalization, the patient was empirically treated with a full course of piperacillin/tazobactam (3.375 g IV every 6 h), metronidazole (400 mg IV every 8 h), vancomycin (1 g every 12 h), and levofloxacin (750 mg IV every 24 h) for 14 days. Additionally, the patient underwent laparotomy, segmental bowel resection, and fascial repair using tension sutures and electrolysis. The patient underwent extubation twice but was reintubated due to respiratory distress and tachypnea, receiving care from the pulmonary service. On day 21 of her ICU stay, due to increased lung secretions, leukocytosis (24,800 per µL) and fever (39°C), a tracheal culture (TC) sample was sent from the patient. The culture result showed growth of CRKP isolate (minimum inhibitory concentrations [MICs] ≥ 32, and < 1 μg/ml for meropenem and colistin, respectively) that was also resistant to all tested antibiotics. According to the result of microbial culture, colistin was prescribed to the patient in two forms, injection (4.5 million IU every 12 h) and nebulizer (1 million IU every 12 h). The patient was continued on colistin for 14 days. Also, simultaneously with the administration of colistin, rifampin (10 mg/kg once daily, 10 days) was also started for the patient. Two days after the duration of treatment with colistin, a TC culture was sent from the patient, which was negative. The patient was discharged from the hospital on day 46.

## Materials and Methods

### Antimicrobial susceptibility testing

The antibiotic susceptibility of two *K. pneumoniae* isolates was determined by Kirby-Bauer disk diffusion assay according to Clinical Laboratory Standards Institute guidelines [6]. Minimum inhibitory concentration (MIC) against meropenem and colistin (Sigma Aldrich Chemicals Pvt. Ltd,India) was determined by microdilution broth method. *Escherichia coli* ATCC 25922 was used as quality control.

### String test

String test was performed on the blood culture to identify the hypermucoviscous phenotype, and a positive result was defined by the formation of viscous strings > 5 mm in length [7].

### Molecular detection of carbapenemase and ESBL-encoding genes

The presence of ESBLs genes (*bla*_CTX-M_, *bla*_TEM_, *bla*_SHV_) and carbapenemases (*bla*_KPC_, *bla*_VIM_, *bla*_IMP_, *bla*_NDM_ and *bla*_OXA-48_) genes were investigated by polymerase chain reaction (PCR) as previously described [5].

### Determination of capsular serotypes, virulence Genes

The genotypes of capsular serotype in K1, K2, K5, K20, K54, and K57 and virulence-associated genes, including *iucA*, *peg-344*, *iutA*, *iroB*, *magA*, *rmpA* and *alls*. were determined using PCR [8, 9].

### Multilocus sequence typing

A multilocus sequence typing (MLST) analysis of the isolated strains was performed with PCR amplification, as previously described. House-keeping genes, including *gapA*, *infB*, *mdh*, *pgi*, *phoE*, *rpoB*, and *tonB*, were sequenced and compared with the MLST allele profiles available at http://www.pasteur.fr/mlst.

## Results

In this study, both patients were admitted to a shared ICU in a university hospital. As shown in Table 1, both isolates were remarkably resistant to almost all tested antimicrobial agents. The MIC results showed that the NO1 isolate was intermediate to colistin and the NO2 isolate was susceptible. Also, both isolates were reported to be susceptible to tigecycline. Analysis of resistance determinants revealed that NO1 isolate carried the *bla*_NDM_, *bla*_OXA-48-like_, *bla*_CTX-M_, *bla*_TEM_, *bla*_SHV_ while NO2 isolate was positive for *bla*_NDM_, *bla*_OXA-48-like_, *bla*_SHV_.

**Table 1:** Microbiological and genomic characteristics of the two carbapenem-resistant K. pneumonia isolates

Patients	Strains	Specimen	String test	Capsular serotype	Virulence genes	MLST	β-lactamase genes	Resistance profile	MIC of antimicrobials (µg/ml)	
									**MEM**	**COL**
Case-1	NO1	Urine	Negative	Neg	Neg	ST16	*bla*_CTX-M_, *bla*_TEM_, *bla*_SHV_, *bla*_NDM_, *bla*_OXA-48-like_	CAZ, CPM, PTZ, AM, GM, CIP, LEV, NIT	16	2
Case-2	NO2	Tracheal	Positive	K2	*iucA*, *rmpA2, rmpA*	ST337	*bla*_SHV_, *bla*_NDM_, *bla*_OXA-48-like_	CAZ, CPM, PTZ, AM, GM, CIP, LEV	≥ 32	< 1

*Neg* negative, *MEM* meropenem, *COL* colistin, *CAZ* ceftazidime, *CPM* cefepime, *PTZ* piperacillin/tazobactam, *AM* amikacin, *GM* gentamicin, *CIP* ciprofloxacin, *LEV* levofloxacin, *NIT* nitrofurantoin

MLST analysis showed that NO1 and NO2 strains belonged to ST16 and ST337, respectively. Only, ST337 isolate demonstrated hypermucoviscosity by string length > 5 mm in the string test. The serotype of the capsule was K2. This strain was positive for virulence genes *iucA*, *rmpA2* and *rmpA*. ST16 strain was negative for virulence and capsular serotype genes.

## Discussion

CRKP is one of the most common carbapenem-resistant Gram-negative pathogens associated with clinical infections such as ventilator-associated pneumonia (VAP), urinary tract infection (UTI) and wound infection in the healthcare setting [10]. Colistin is one of the last resort antimicrobial agents for the treatment of infections caused by these pathogens. However, worldwide rates of colistin resistance varied between 2.8 and 10.5% for *K*. *pneumonia* over the last decade [11]. In Iran, because the only choice for treating patients infected with these strains in most hospitals is colistin there is a concern that the rate of resistance to this antibiotic will increase very quickly. In recent years, there has been an increase in Enterobacterales that co-produce two or more types of carbapenemases, and this poses a much greater public health risk. Previous studies in Iran showed that this type of isolate with two carbapenemases is frequent, especially among CRKP isolates [5, 12]. The increase in the prevalence of carbapenemase-producing isolates may lead to the need to use more colistin in our country.

In previous studies the spread of CRKP ST16 co-harboring both *bla*_NDM-1_ and *bla*_OXA-48_ has been reported, calling for careful monitoring of these highly resistant clones [13, 14]. Consistent with our research, ST16 *K. pneumoniae* often carried ESBL and carbapenemase genes such as *bla*_CTX-M,_* bla*_NDM_*,* and *bla*_OXA-48_*.* Similar to a previous report in Thailand [13], hypervirulence genes (*iucA*, *peg-344*, *iutA*, *iroB*, *magA*, *rmpA* and *alls*) were not identified in the ST16 isolate in the current study. This strain was phenotypically intermediate to colistin with a MIC value of 2 µg/ml, according to the CLSI recommendations. Since the physician had no choice but to use colistin, a combination treatment experimentally of meropenem (without determining the MIC) with colistin was started for the patient's UTI.

Among two CRKP isolates, only the isolate that recovered from the TC sample of case 2 was positive for virulence-determinant genes *iucA*, *rmpA2 and rmpA* and capsular genes of K2 corresponding to invasive *K. pneumoniae* ST337 clone producing *bla*_NDM_ and *bla*_OXA-48like_.

The outbreak of *bla*_NDM-5_-carrying ST337 CRKP was identified from neonates in previous studies [15, 16], however, no report has described NDM-1 and OXA-48-producing CRKP ST337 clone. This is the first identification of harbouring *bla*_NDM_ and *bla*_OXA-48-like_ CRKP strain belonging to hypervirulent ST337. This highlights that CRKP isolates with *bla*_NDM_ and *bla*_OXA-48-like_ genes continue to be a problem in Iran, especially in VAP in ICUs. In this study, case 2 was intubated twice which can cause lung infection. According to a previous study, reintubation is associated with increases in the likelihood of VAP [17].

In this study, the first patient was treated with the combination of colistin plus meropenem, while the second patient was treated with the combination of colistin plus rifampin. Previous studies have shown that colistin combination therapy with other antibiotics has the potential as a treatment option for infection with CRKP. Therefore, colistin-meropenem combination may be an appropriate option if the MIC is ≤ 32 mg/L [18, 19]. According to previous reports in Iran, there is a possibility that NDM-1 and OXA-48-producing CRKP ST337 clone may be a new clone in our country. While ST16 has been previously reported in Iran and seems to be an endemic strain in Iran, it should be noted that our previous study showed that clone ST16 is a high-risk endemic clone in our hospital [12].

Considering the prevalence of carbapenemases in both isolates it is expected that both strains were resistant to the β-lactam/β-lactamase inhibitor combinations. However, cefiderocol could be an alternative to colistin, but a limitation of our study is not having tested these antibiotics.

This study has some limitations. First, due to the limited capacity of the laboratory and lack of budget, we could not use techniques such as whole-genome sequencing for further genomic investigations. Second, due to the lack of innovative β-lactam/β-lactamase inhibitor combinations discs in Iran, we could not check the antibiotic resistance profile of these drugs for CRKP isolates.

In conclusion, to the best of our knowledge, this case is the first report of K2-ST337 hvKP coproducing *bla*_NDM_ and *bla*_OXA-48-like_ isolate causing VAP. Due to the high prevalence of CRKP strains in Iran, some of which are hypervirulent, colistin is currently being prescribed a lot in our country. Increasing the use of colistin for multidrug-resistant Gram-negative bacterial infections will lead to the emergence of colistin resistance. Therefore, infection control programs including hand hygiene, outbreak management, environmental hygiene, surveillance, education, isolation, infection prevention policies and management should be important to prevent and control the spread of these bacteria in hospitals.

## Data Availability

All data are available within the manuscript.

## Abbreviations

CRKP: Carbapenem-resistant *Klebsiella pneumonia*
UTI: Urinary tract infection
VAP: Ventilator-associated pneumonia
cKP: Classic *K. pneumonia*
hvKPs: Hypervirulent *K. pneumonia*
ESBL: Extended-spectrum β-lactamase
ICU: Intensive care unit
MICs: Minimum inhibitory concentrations
TC: Tracheal culture
PCR: Polymerase chain reaction
MLST: Multilocus sequence typing
